# Visit-to-visit glycemic variability is associated with lung function variables and lung function impairment in individuals with type 2 diabetes

**DOI:** 10.1371/journal.pone.0337885

**Published:** 2025-12-01

**Authors:** Yi-Hua Wu, Chia-Ing Li, Chiu-Shong Liu, Chih-Hsueh Lin, Shing-Yu Yang, Cheng-Chieh Lin, Tsai-Chung Li

**Affiliations:** 1 Center for Digestive Medicine, Department of Internal Medicine, China Medical University Hospital, Taichung, Taiwan; 2 Taiwan Association for the Study of Intestinal Diseases, Taoyuan, Taiwan; 3 School of Medicine, College of Medicine, China Medical University, Taichung, Taiwan; 4 Department of Medical Research, China Medical University Hospital, Taichung, Taiwan; 5 Department of Family Medicine, China Medical University Hospital, Taichung, Taiwan; 6 Department of Public Health, College of Public Health, China Medical University, Taichung, Taiwan; 7 Department of Audiology and Speech-Language Pathology, Asia University, Taichung, Taiwan; Tecnologico de Monterrey, MEXICO

## Abstract

Glycemic variability (GV) is an emerging biomarker of glycemic control and may be a predictor for lung function impairment in persons with type 2 diabetes mellitus (T2DM). However, the associations between GV and lung function variables and lung function impairment have not been fully evaluated. The objective of this study was to assess the associations of glycemic variability (GV) with lung function impairment in persons with T2DM. A follow-up study was conducted on the data of 3,108 subjects collected from 2001 to 2020 using the diabetes care management program database in Taiwan. GV in fasting plasma glucose (FPG) was calculated using standard deviation (SD), average real variability (ARV), coefficient of variation (CV), variability independent of the mean (VIM), and slope of 1-year repeated measurements. A ratio of forced expiratory volume in 1 s (FEV1) to forced vital capacity (FVC) less than 0.70 was used to define lung function impairment. Multivariable linear and logistic regression models were applied to explore the relationships of GV with lung function variables and lung function impairment. A total of 359 (11.6%) subjects were defined as having lung function impairment. After multivariable adjustment, FPG‐SD, FPG-CV, FPG-AVR, FPG-VIM and were found to be negatively linked with FEV1, % predicted FEV1, and FVC but not FEV1/FVC. Relative to those for the first tertile, the odds ratios (ORs) of lung function impairment for the second and third tertiles were 1.37 (95% confidence interval [CI]: 1.01, 1.87) and 1.51 (1.10, 2.08) for FPG-CV, respectively; 1.59 (1.16, 2.17) and 1.73 (1.24, 2.40) for FPG‐SD, respectively; and 1.57 (1.15, 2.13) and 1.69 (1.22, 2.33) for FPG-AVR, respectively. GV, measured by CV, SD, VIM, and VIM, is linked with lung function impairment and all lung function variables, except for FEV1/FVC ratio. GV may serve as a useful biomarker for assessing lung function impairment in persons with T2DM.

## Introduction

Diabetic microangiopathy affects the lungs as it does other organs; however, the recognition of the lungs as a target of diabetes is relatively recent. Individuals with type 1 diabetes exhibit reduced total lung capacity and indicators of pulmonary restrictive dysfunction compared with their nondiabetic peers [1]. A systemic review of cross-sectional studies indicated that the lung volumes of persons with type 2 diabetes are 3%–10% lower than those of individuals without diabetes, regardless of body mass index (BMI) and smoking status. However, findings from longitudinal studies are inconsistent. Some reported that individuals with type 2 diabetes experience an accelerated decline in lung function over time [2,3], but others did not observe such decline [4,5].

Pulmonary function is most commonly assessed using the noninvasive technique of spirometry [6]. Key indicators such forced vital capacity (FVC), forced expiratory volume in 1 second (FEV1), and FEV1/FVC ratio are crucial measurements for assessing lung function. FEV1, which indicates the volume of air exhaled in the first second of a forced breath, is critical for evaluating airflow obstruction. FVC measures the total volume of air exhaled forcefully after a full inhalation and is used to assess lung volume. The FEV1/FVC ratio, whose low value indicates obstruction, helps differentiate between restrictive and obstructive (such as chronic obstructive pulmonary disease, chronic obstructive pulmonary disease [COPD] or asthma) lung diseases. The importance of these lung function measures lies in their ability to facilitate the early diagnosis of respiratory disease and condition, track disease progression to therapy, and ultimately improve management for individuals with respiratory conditions.

In addition to single glucose measurements (glycated hemoglobin [HbA1c] or fasting plasma glucose [FPG]), glycemic variability has emerged as a significant factor in blood glucose regulation and a key target in diabetes care. Growing body of evidence suggests that glucose fluctuations may serve as a novel predictor of mortality and microvascular and macrovascular morbidity [7–12]. A previous study assessed the association between glycemic variability and COPD risk in individuals with type 2 diabetes by utilizing International Classification of Disease, 9th Revision, Clinical Modification (ICD-9-CM) codes from insurance claims data [13]. However, the relationship between glycemic variability and lung function variables or lung function impairment as determined by spirometry measurements has never been explored.

## Materials and methods

### Study design and criteria for study participants

This retrospective cohort study examined the enrollees of the Diabetes Care Managed Program (DCMP), which has been operational since November 2001. The DCMP aims to enhance quality of diabetes care and reduce complications via close monitoring, continuous support, and promotion of healthy lifestyle choices. Study subjects were enrolled during the period from November 2001 through December 2020. Eligibility criteria were as follows: diabetes diagnosis (ICD-9-CM code 250 prior to 2016 or International Classification of Disease, Tenth Revision, Clinical Modification [ICD-10-CM] code E0800 thereafter), aged 30 years and above, and completion of lung function examination. Initially, 4,128 individuals were enrolled at a teaching medical center in Taiwan. The exclusion criteria were as follows: type 1 diabetes (ICD-9-CM codes 250.x1/x3; ICD-10-CM code E10.9), ICD-10-CM code O24.419), pregnancy-associated diabetes (ICD-9-CM code 648.83; age under 30 years, and no data for glycemic variability (n = 986). After these exclusions were applied, 3,142 participants remained. Further exclusions due to data attrition on anthropometric measurements, comorbidities, and biochemical tests lowered the final sample size to 3,108 study subjects for analysis (S1 Fig). The reference date for each participant was the date of their first lung function assessment; when multiple assessments were conducted, only the first examination was considered. Measurements for other variables were collected on or near the reference date, and glycemic variability measures were derived from data gathered in the year prior to the reference date. Approval for this study was granted by the Research Ethics Committee in China Medical University Hospital (CMUH), and all procedures adhered to the relevant guidelines and regulatory standards. The Research Ethics Committee granted a waiver of inform consent (CMUH112-REC2–180), because the dataset employed in this study comprised de-identified secondary data made available for research.

### Data source

Data were sourced from the electronic database of the DCMP at CMUH in Taichung, Taiwan. This program includes persons with type 2 diabetes diagnosed in accordance with the American Diabetes Association guidelines. Healthcare providers involved in the DCMP take part in training and clinical education activities to enhance their skills. The healthcare team is composed of specialists from various fields, including family medicine, endocrinology, cardiology, internal medicine, and nephrology. Ongoing education and training within the DCMP aim to standardize clinical practices related to assessing and tracking blood sugar levels and diagnosing complications of diabetes. Collaborative care is delivered by doctor-led interdisciplinary teams, which include doctors and case managers who adhere to evidence-based diabetes care management. The DCMP database contains comprehensive diabetes care information including yearly self-care assessments and instruction, yearly eye examinations, and quarterly screenings for cholesterol, blood sugar, and kidney function.

### Measurements

Prior to enrolling in the DCMP, the study subjects underwent urine, blood, and body measurements and provided responses in interviews by a case manager using a standardized electronic questionnaire. This assessment was conducted annually or quarterly to obtain information about lifestyle, dietary habits, and medical history, including current or previous diseases. Sociodemographic information included age at entry, sex, and family medical history of hypertension, diabetes, obesity, and hyperlipidemia. Assessed lifestyle behaviors included alcohol consumption, smoking status, and leisure-time physical activity, which were categorized according to study subjects’ self-reports (yes versus no).

Baseline coexisting conditions included hypertension, hyperlipidemia, and obesity. Complications of diabetes were grouped into chronic and acute categories. Chronic complications encompassed peripheral vascular disease, neuropathy, stroke, nephropathy, amputation, diabetic retinopathy, and diabetic foot. Acute complications consisted of diabetic ketoacidosis, hypoglycemia, and hyperosmolar nonketotic coma. Coexisting conditions and complications were documented using a yes/no classification.

Diabetes-related variables included duration of diabetes condition. Anti-diabetes medication was classified into two main categories: insulin therapy and oral drugs. Oral drugs were classified into 7 classes: sulfonylureas, meglitinides, biguanides, dipeptidyl peptidase 4 inhibitors, α-glucosidase inhibitors, other compounds, and insulin sensitizers. Pharmacological variables encompassed drugs for hyperlipidemia (e.g., statins [HMG-CoA reductase inhibitors]), elevated blood pressure (e.g., calcium channel blockers), and nephropathy. The participants were categorized according to drug uses as recorded in their computerized medical records (yes or no).

Blood specimens were collected via antecubital venipuncture during morning hours following a 12-hour period of fast and processed within four hours after sample collection. Biochemical tests included the measurement of FPG, HbA1c, total cholesterol (TC), serum creatinine, serum low-density lipoprotein cholesterol (LDL-C), triglycerides (TG), high-density lipoprotein cholesterol (HDL-C), and urinary protein concentration. A biochemical auto-analyzer (Beckman Coulter Synchron system, Lx-20, Fullerton, CA, USA) was used to analyze these biochemical markers. The estimated glomerular filtration rate (eGFR) was derived from serum creatinine measurements using the Chronic Kidney Disease Epidemiology Collaboration formula [14].

Anthropometric measurements included height, weight, and BMI. Height and weight were recorded with an automated anthropometric device (Super-view HW-666); the study subjects were instructed to take off their shoes and dress in light attire. BMI was derived using the formula: weight (kg)/ (height (m)².

Glycemic variability evaluated by FPG variability were calculated as coefficient of variation (CV), variability independent of the mean (VIM), standard deviation (SD), average real variability (ARV), and slope. To account for considering for the potential impact of varying numbers of measurements per individual, the CV is divided by $\sqrt{n/(n-\text{1})}$, as recommended from prior study [15]. These variables were calcaulated as observing at least two measurements of FPG in one-year intervals.

### Outcome measures

A spirometry test (EasyOne spirometer; NDD [New Diagnostic Design], Zurich, Switzerland) was conducted to measure pulmonary function by qualified and trained nurses following a standardized protocol [16]. Spirometry evaluates the coordinated mechanical performance of the chest wall, lungs, airways, and respiratory muscles by assessing the total exhaled air volume forcefully from fully inflated lungs to full expiration. During the test, the study subjects sat down and fitted with a nose clip. They were directed to take a deep breath, followed by a brief pause (<1 s), and exhale fully until no additional air could be released while remaining upright. Each subject underwent a minimum of three tests, and the highest values for FEV1, % predicted FEV1, FEV, and FEV1/FVC ratio were recorded for analysis.

For the measurements to be considered repeatable, the two largest values for FVC and FEV1 must differ by no more than 0.150 L, unless the maximum measurement for either parameter falls short of 1 L. FVC represents the full amount of air expelled during a forceful exhalation after the deepest possible breath. FEV1 refers to the air volume breathed out in the initial second of a forced exhale. The % predicted FEV1 was derived by expressing the patient’s FEV1 as a ratio of the expected value for a healthy individual with comparable sex, age, and body characteristics. The FEV1/FVC ratio represents the fraction of the FEV1 relative to FVC. An FEV1/FVC ratio less than 0.7 was used to indicate impaired lung function [17].

### Statistical analysis

Descriptive statistics such as standard deviations, and means were summarized as continuous variables. Meanwhile, proportions and frequencies were used for categorical variables. An independent t-test was applied to continuous variables and Chi-square tests to categorical variables for bivariate analyses. The following steps were conducted to build linear models with multiple predictors: first, univariate models were built for main exploratory variables (glucose variability in HbA1c and FPG) and all covariates, and variables with p-values <0.25 were selected for further analysis [18,19]. Next, glucose variability in HbA1c and FPG were included one at a time to multiple models with the selected covariates from the previous process to assess their statistical significance (p-value <0.05). Regression coefficients were subsequently estimated using multiple linear regression models. SAS version 9.4 (SAS, Cary, NC) was used for all analyses, with two-tailed p-values and significance set at p < 0.05.

## Results

Among the 3,108 persons with type 2 diabetes, the average age was 63.1 years with a SD of 11.2. Among these participants, 1,920 (61.8%) were men. Table 1 shows that individuals with lung function impairment had a significantly higher age (p < 0.001), had a higher proportion of smoking (p = 0.02), and had a reduced mean BMI (p < 0.001) than those without. In addition, the men exhibited lower prevalence of hyperlipidemia (p = 0.005) and obesity (p = 0.02) compared with the women. Furthermore, individuals with lung function impairment had a higher likelihood of using cardiovascular medication (p = 0.03) than those without.

**Table 1:** Comparison of sociodemographic factors, diabetes-related variables, lifestyle behaviors, biomarkers, and comorbidities between individuals with and without lung function impairment.

Variables	Total	Lung function impairment, N (%)		P value
	(*n* = 3,108)	No (*n* = 2,749)	Yes (*n* = 359)	
**Sociodemographic factors**				
Age, years^†^	63.09 ± 11.18	62.22 ± 11.04	69.78 ± 9.89	<0.001
Gender				<0.001
Men	1920 (61.78)	1653 (60.13)	267 (74.37)	
Women	1188 (38.22)	1096 (39.87)	92 (25.63)	
**Lifestyle behaviors**				
Smoking				0.02
No	2545 (81.89)	2267 (82.47)	278 (77.44)	
Yes	563 (18.11)	482 (17.53)	81 (22.56)	
Alcohol drinking				0.78
No	2867 (92.25)	2534 (92.18)	333 (92.76)	
Yes	241 (7.75)	215 (7.82)	26 (7.24)	
Physical activity				0.06
No	1645 (52.93)	1438 (52.31)	207 (57.66)	
Yes	1463 (47.07)	1311 (47.69)	152 (42.34)	
BMI, kg/m^2^^†^	26.22 ± 4.25	26.39 ± 4.29	24.94 ± 3.71	<0.001
**Diabetes-related variables**				
Duration of diabetes, years^†^	6.14 ± 7.23	6.06 ± 7.19	6.75 ± 7.47	0.09
Type of hypoglycemic drug use				0.84
No	207 (6.66)	182 (6.62)	25 (6.96)	
OAD	2452 (78.89)	2169 (78.9)	283 (78.83)	
Inject insulin	62 (1.99)	57 (2.07)	5 (1.39)	
Both	387 (12.45)	341 (12.4)	46 (12.81)	
**Comorbidity**				
Hypertension	1084 (34.88)	962 (34.99)	122 (33.98)	0.75
Hyperlipidemia	744 (23.94)	680 (24.74)	64 (17.83)	0.005
Obesity	738 (23.75)	671 (24.41)	67 (18.66)	0.02
Coronary artery disease	221 (7.11)	195 (7.09)	26 (7.24)	1.00
Stroke	78 (2.51)	67 (2.44)	11 (3.06)	0.59
Peripheral neuropathy	209 (6.72)	187 (6.8)	22 (6.13)	0.71
Neuropathy	54 (1.74)	50 (1.82)	4 (1.11)	0.46
Nephropathy	155 (4.99)	139 (5.06)	16 (4.46)	0.72
**Drug-related variables**				
Hypertension medications	1126 (36.23)	991 (36.05)	135 (37.6)	0.60
Hyperlipidemia medications	524 (16.86)	471 (17.13)	53 (14.76)	0.29
Cardiovascular medications	713 (22.94)	614 (22.34)	99 (27.58)	0.03
**Biomarker^†^**				
FPG (mg/dl)	141.26 ± 50.96	141.57 ± 51.39	138.87 ± 47.52	0.34
HbA1c (%)	7.61 ± 1.54	7.62 ± 1.56	7.54 ± 1.44	0.36

Student’s t tests were used to assess the differences in continue variables while Chi-square tests were applied to categorical variables.

HbA1c: Hemoglobin A1c; FPG: Fasting plasma glucose.

^†^: data are reported as mean ± standard deviation (SD).

**Fig 1:**
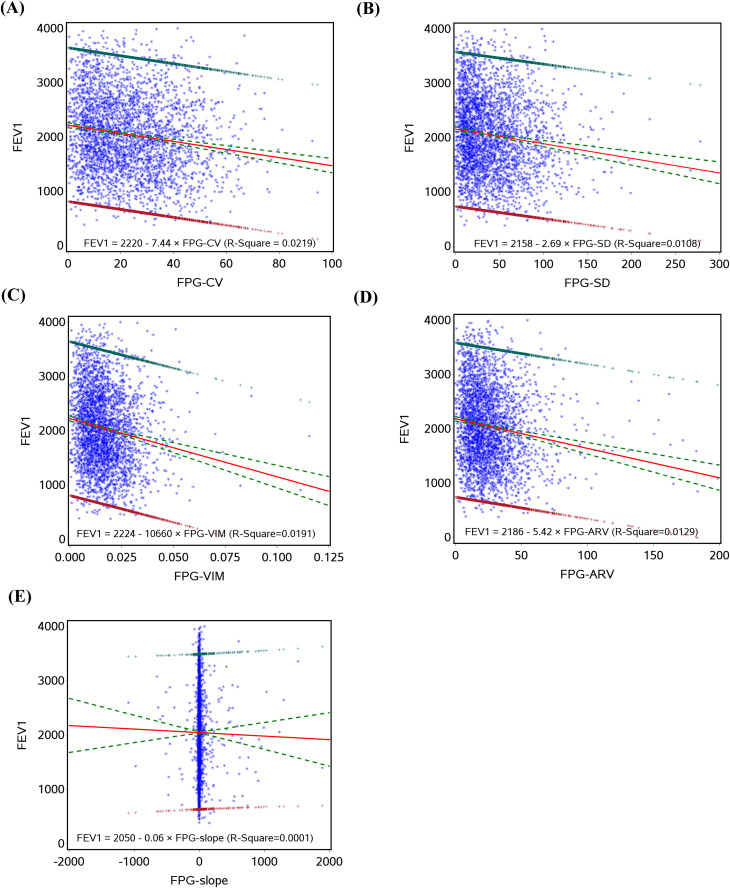
The linear regression with FEV1, regressed on FPG-CV (A), FPG-SD (B), FPG-VIM (C), FPG-ARV (D), and FPG-slope (E).

**Fig 2:**
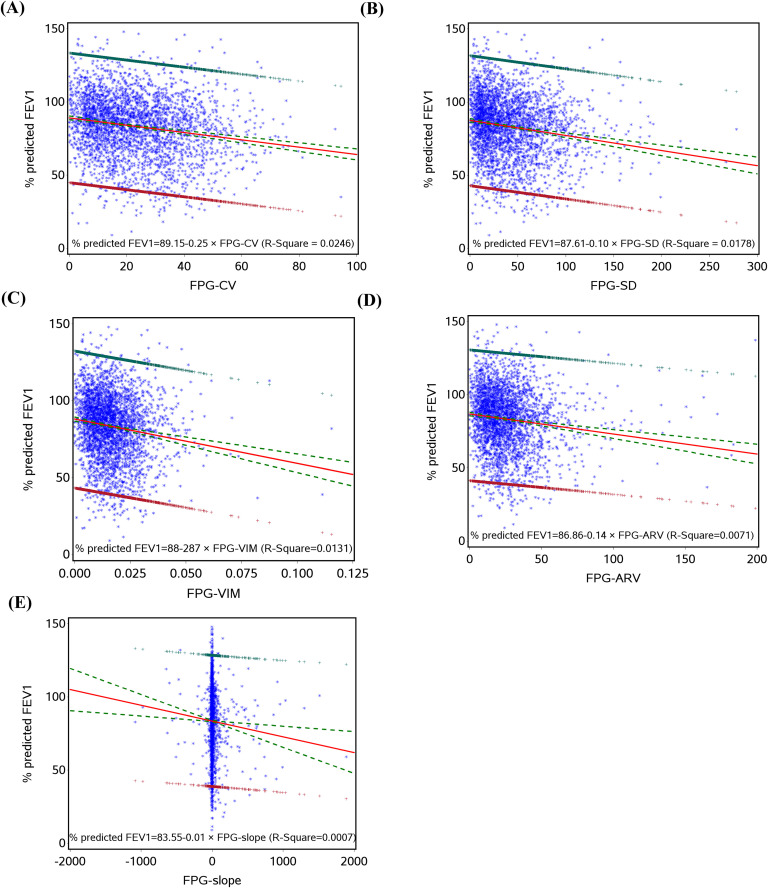
The linear regression with % predicted FEV1, regressed on FPG-CV (A), FPG-SD (B), FPG-VIM (C), FPG-ARV (D), and FPG-slope (E).

**Fig 3:**
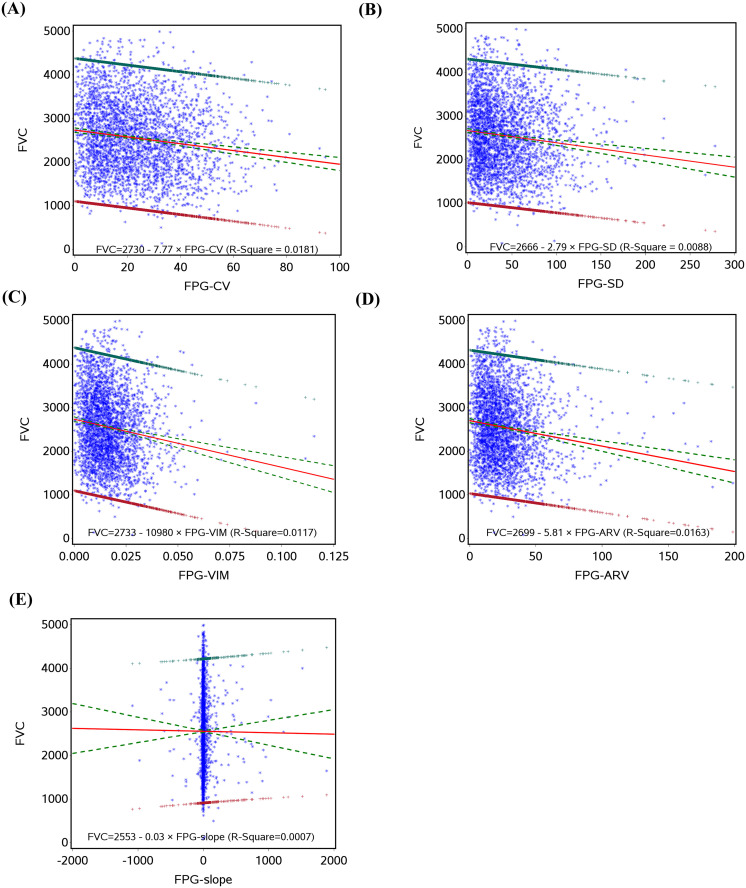
The linear regression with FVC, regressed on FPG-CV (A), FPG-SD (B), FPG-VIM (C), FPG-ARV (D), and FPG-slope (E).

Prior to fitting the linear regression between glucose variation and lung function variables, their relationships were assessed and found to be linear as shown in Figs 1–3. Table 2 presents the relationships between glucose variation and lung function variables FEV1, % predicted FEV1, FEV1/FVC, and FVC. In models adjusted for age and sex, all FPG variation variables were significantly linked with FEV1, except for FPG-slope. These relationships continued to be significant after controlling for lifestyle behaviors and baseline blood glucose. Further controlling for coexisting conditions and drug use did not alter these significant relationships. These results indicate a negative association, meaning that great glucose variation values are linked to low FEV1 [FPG-CV (b=−76.66, p < 0.001), FPG-SD (b=−66.88, p < 0.001), FPG-VIM (b=−52.05, p < 0.001), and FPG-ARV (b=−38.91, p < 0.001)]. Similarly, glucose variation variables (except FPG-slope) were significantly associated with %predicted FEV1 [FPG-CV (b=−2.98, p < 0.001), FPG-SD (b=−2.65, p < 0.001), FPG-VIM (b=−1.98, p < 0.001), and FPG-ARV (b=−1.24, p < 0.001)] and FVC [FPG-CV (b=−84.76, p < 0.001), FPG-SD (b=−73.43, p < 0.001), FPG-VIM (b=−57.38, p < 0.001), and FPG-ARV (b=−43.08, p < 0.001)]. None of the glucose variation variables were significantly associated with FEV1/FVC.

**Table 2:** Relationship of glycemic variability with lung function in persons with type 2 diabetes.

Lung function	Glucose variation per 1 SD									
	FPG-CV		FPG-SD		FPG-VIM		FPG-ARV		FPG-slope	
	β (SE)	R^2^	β (SE)	R^2^	β (SE)	R^2^	β (SE)	R^2^	β (SE)	R^2^
FEV1										
Model 1	−87.05 (9.36)***	0.49	−75.52 (9.36)***	0.49	−63.05 (9.43)***	0.49	−54.46 (9.42)***	0.48	−10.82 (9.45)	0.48
Model 2	−77.13 (10.33)***	0.50	−66.25 (10.71)***	0.50	−53.44 (9.65)***	0.50	−40.6 (10.03)***	0.49	−6.94 (9.37)	0.49
Model 3	−76.66 (10.41)***	0.51	−66.88 (10.78)***	0.50	−52.05 (9.68)***	0.50	−38.91 (10.05)***	0.50	−9.19 (9.37)	0.50
% predicted FEV1										
Model 1	−3.36 (0.40)***	0.04	−2.92 (0.40)***	0.03	−2.43 (0.41)***	0.03	−1.86 (0.41)***	0.02	−0.60 (0.41)	0.02
Model 2	−2.99 (0.45)***	0.05	−2.61 (0.46)***	0.04	−2.04 (0.42)***	0.04	−1.31 (0.43)**	0.04	−0.48 (0.41)	0.04
Model 3	−2.98 (0.45)***	0.06	−2.65 (0.47)***	0.05	−1.98 (0.42)***	0.05	−1.24 (0.44)**	0.04	−0.54 (0.41)	0.04
FEV1/FVC										
Model 1	−0.41 (0.72)	0.01	−0.56 (0.72)	0.01	0.39 (0.72)	0.01	0.14 (0.72)	0.01	−0.34 (0.72)	0.01
Model 2	0.20 (0.80)	0.01	−0.02 (0.83)	0.01	0.81 (0.75)	0.01	0.61 (0.77)	0.01	−0.26 (0.72)	0.01
Model 3	0.16 (0.81)	0.01	−0.07 (0.84)	0.01	0.79 (0.75)	0.01	0.59 (0.78)	0.01	−0.28 (0.73)	0.01
FVC										
Model 1	−95.78 (10.82)***	0.49	−82.56 (10.82)***	0.49	−69.32 (10.89)***	0.49	−60.25 (10.87)***	0.48	−3.69 (10.92)	0.48
Model 2	−85.69 (11.93)***	0.50	−73.03 (12.36)***	0.50	−59.07 (11.15)***	0.50	−45.42 (11.57)***	0.50	0.02 (10.82)	0.49
Model 3	−84.76 (12.01)***	0.51	−73.43 (12.44)***	0.50	−57.38 (11.17)***	0.50	−43.08 (11.59)***	0.50	−2.34 (10.81)	0.50

Multivariate model 1 controlled for sex and age.

Multivariate model 2 controlled for diabetes-related variables, life style behaviors, FPG and HbA1c in addition to sex and age in multivariate model 1.

Multivariate model 3 controlled for baseline status of medicines use and complications in addition to the variables in the multivariate model 2.

*: p < 0.05; **:p < 0.01; ***:p < 0.001.

The ORs and 95% CI estimated from the three lung function impairment models based on glycemic variability measures are presented in Table 3. FPG-SD, FPG-CV, and FPG-ARV were significant in the model controlled for age and sex (model 1). They remained significant after accounting for diabetes-related factors, lifestyle behaviors, FPG, and HbA1c (model 2) and after further adjusting for the baseline status of complications and medication use (model 3). The ORs of lung function impairment for the second and third tertiles of FPG-CV in model 3 were 1.37 (95% CI: 1.01, 1.87, p < 0.05) and 1.51 (1.10, 2.08, p < 0.05), respectively; for FPG-SD, they were 1.59 (1.16, 2.17, p < 0.01) and 1.73 (1.24, 2.40, p < 0.01), respectively; and for FPG-ARV, they were 1.57 (1.15, 2.13, p < 0.01), and 1.69 (1.22, 2.33, p < 0.01), respectively. The linear trends of FPG-CV and FPG-SD were significant [per 1 SD: 1.15 (1.02, 1.30, p < 0.05) and 1.18 (1.04, 1.32, p < 0.01), respectively], but the effect of FPG-ARV was not significant.

**Table 3:** Association of glycemic variability with lung function impairment in persons with type 2 diabetes.

		Lung function impairment (FEV1/FVC < 70%) OR (95% CI)		
Glucose variability	n	Model 1	Model 2	Model 3
FPG-CV (per 1 SD)	3108	1.15 (1.03, 1.28)*	1.14 (1.01, 1.28)*	1.15 (1.02, 1.30)*
Tertile 1	1026	1.00	1.00	1.00
Tertile 2	1025	1.34 (0.99, 1.80)	1.35 (1.00, 1.84)	1.37 (1.01, 1.87)*
Tertile 3	1057	1.48 (1.11, 1.97)**	1.47 (1.07, 2.02)*	1.51 (1.10, 2.08)*
FPG-SD (per 1 SD)	3108	1.17 (1.06, 1.29)**	1.16 (1.04, 1.31)*	1.18 (1.04, 1.32)**
Tertile 1	1025	1.00	1.00	1.00
Tertile 2	1026	1.51 (1.12, 2.03)**	1.56 (1.14, 2.12)**	1.59 (1.16, 2.17)**
Tertile 3	1057	1.65 (1.23, 2.21)***	1.70 (1.22, 2.36)**	1.73 (1.24, 2.40)**
FPG-VIM (per 1 SD)	3108	1.11 (0.99, 1.23)	1.10 (0.98, 1.23)	1.10 (0.99, 1.24)
Tertile 1	1026	1.00	1.00	1.00
Tertile 2	1025	1.07 (0.80, 1.44)	1.07 (0.79, 1.45)	1.09 (0.81, 1.48)
Tertile 3	1057	1.31 (0.99, 1.73)	1.29 (0.96, 1.72)	1.31 (0.98, 1.77)
FPG-ARV (per 1 SD)	3108	1.11 (1.01, 1.22)*	1.10 (0.99, 1.23)	1.10 (0.99, 1.23)
Tertile 1	1026	1.00	1.00	1.00
Tertile 2	1025	1.54 (1.14, 2.08)**	1.54 (1.13, 2.09)**	1.57 (1.15, 2.13)**
Tertile 3	1057	1.62 (1.21, 2.16)**	1.66 (1.20, 2.28)**	1.69 (1.22, 2.33)**
FPG-slope (per 1 SD)	3108	1.09 (0.97, 1.23)	1.08 (0.96, 1.21)	1.08 (0.96, 1.22)
Tertile 1	1026	1.00	1.00	1.00
Tertile 2	1026	0.89 (0.67, 1.19)	0.91 (0.68, 1.22)	0.91 (0.68, 1.22)
Tertile 3	1056	1.26 (0.96, 1.66)	1.26 (0.95, 1.67)	1.26 (0.95, 1.67)

Multivariate model 1 controlled for sex and age.

Multivariate model 2 controlled for diabetes-related variables, life style behaviors, FPG and HbA1c in addition to sex and age in multivariate model 1.

Multivariate model 3 controlled for baseline status of medicines use and complications in addition to the variables in the multivariate model 2.

*: p < 0.05; **:p < 0.01; ***:p < 0.001.

## Discussion

This study thoroughly examined the independent relationships between various measures of glycemic variability in FPG with distinct lung function variables and lung function impairment determined by the FEV1/FEV ratio. Except for the FEV1/FVC ratio, all the lung function variables tested were significantly linked with glycemic variability markers in FPG, with the exception of the FPG-slope. The strongest association was observed with FPG-CV, and the weakest was with FPG-ARV. FPG-CV and FPG-SD were the only two measures of variability found to be associated with lung function impairment as determined by the FEV1/FVC ratio after multivariate adjustment.

Prior studies explored the associations between diabetes status and lung function variables using cross-sectional [2,3,5,20,21] or longitudinal designs [3–5] and examined the relationship between glucose measures such as FPG and lung function variables in the general population [22–24] or persons with type 2 diabetes [25]. Most of the cross-sectional studies on the relationships between diabetes status and lung function variables found significant associations [2–5,20,21]. Similarly, most longitudinal studies reported that diabetes status is linked with changes in lung function variables [3,4], with the exception of one research conducted in older adults [5]. The studies examining the associations between blood glucose variables and lung function found that FPG [22,25] was associated with lung function variables. However, none of these studies investigated the relationships between visit-to-visit glycemic variability and lung function markers. Visit-to-visit glycemic variability refers to fluctuations in a person’s blood glucose levels across multiple healthcare visits, typically assessed over months. This metric is important for diabetes care because it provides crucial insights into the stability of a patient’s blood glucose control over time. It may offer an accurate reflection of overall glycemic control and potential risks of hypoglycemia and hyperglycemia. In addition, visit-to-visit glycemic variability is associated with the long-term risk of complications [7,11,12,26].

A potential biological mechanism may explain the link between glycemic variability and lung function impairment. Chronic hyperglycemia can lead to the glycosylation of serum and tissue proteins, resulting in the formation of advanced glycation end-products [27]. Once deposited in tissues, these glycosylated proteins trigger pro-inflammatory responses, contributing to microangiopathic complications, such as damage to the microvascular units of the alveolar–capillary network. In addition, chronic hyperglycemia may stimulate inflammatory responses [28,29], which can ultimately cause structural changes in lung tissue and impair lung function. Systemic and local chronic inflammation likely play a significant role, as hyperglycemia rapidly increases circulating cytokine levels through an oxidative mechanism. In vivo studies showed that chronic hyperglycemia can induce structural changes in lung tissue through oxidative stress, leading to restricted gas exchange [30].

This study had strengths. It was a follow-up study conducted with a hospital sample of participants enrolled in DCMP who regularly had outpatient visits and completed multiple clinical examinations. This approach enabled us to gather extensive data on their lung function variables, comorbidities, medication use, and lifestyle factors, allowing us to adjust for confounding variables effectively. This study also had some limitations. First, as this study is observational in nature, its findings may be affected by potential residual and unrecognized confounding variables. Second, the lung function variables were taken at one point in time, without accounting for trends over time. Measurement errors could have occurred due to the extensive data collected from clinical practice. Third, we had access to 1-year FPC measurements in the DCMP dataset, which restricted our ability to evaluate the impact of FPC-CV on lung function during follow-up. Fourth, the study did not include data on respiratory diseases such as bronchiolitis, asthma, COPD, and lung cancer. As a result, we were unable to assess potential associations between glycemic variability and these conditions. Last, our analysis was based on data from a single hospital, which may limit the external generalizability of the findings to broader populations of individuals with diabetes, particularly those in different geographic or healthcare settings. Nevertheless, the findings remain relevant and applicable to populations with type 2 diabetes who share similar demographic and clinical characteristics to our study cohort.

## Conclusions

Except for the FEV1/FVC ratio, all the lung function variables tested and lung function impairment were significantly linked with glycemic variability measures in FPG, with the exception of the FPG-slope. Understanding the link between glycemic variability and lung function impairment underscores the importance of effectively managing blood glucose levels, particularly in patients with diabetes and respiratory conditions, to prevent or mitigate the deterioration of their lung health.

## Supporting information

S1 Fig Flowchart of recruitment procedure. (DOCX)

## Abbreviation

BMI: Body mass index
FEV1: Forced expiratory volume in 1second
FVC: Forced vital capacity
COPD: Chronic obstructive pulmonary disease
HbA1c: Glycated hemoglobin
FPG: Fasting plasma glucose
ICD-9-CM: International Classification of Disease, 9th Revision, Clinical Modification
DCMP: Diabetes Care Management Program
ICD-10-CM: International Classification of Disease, Tenth Revision, Clinical Modification
CMUH: China Medical University Hospital
TC: Total cholesterol
LDL-C: low-density lipoprotein cholesterol
TG: Triglycerides
HDL-C: High-density lipoprotein cholesterol
eGFR: stimated glomerular filtration rate
CV: Coefficient of variation
SD: Standard deviation
VIM: Variability independent of the mean
ARV: Average real variability
