# Supplementary material for: Visit-to-visit glycemic variability is associated with lung function variables and lung function impairment in individuals with type 2 diabetes
Source: PLoS One. 2025 Dec 1;20(12):e0337885. doi: 10.1371/journal.pone.0337885 (PMC12668478; doi:10.1371/journal.pone.0337885)
Supplement: S1 Fig — (DOCX) [file pone.0337885.s001.docx]

34 No data for sociodemographic factors, life style

behaviors, diabetes-related variables, comorbidity

and drug-related variables

4,128 persons enrolled in the Taiwan National Diabetes Care Management Program (NDCMP) with pulmonary function testing

3,142 subjects were eligible

986 Total number of subjects being excluded

37 With type 1 diabetes

34 Age < 30 years

915 No data for glycemic variability

3,108 participants were included for analysis

S1 Fig. Flowchart of recruitment procedure.
